# Image Turing test and its applications on synthetic chest radiographs by using the progressive growing generative adversarial network

**DOI:** 10.1038/s41598-023-28175-1

**Published:** 2023-02-09

**Authors:** Miso Jang, Hyun-jin Bae, Minjee Kim, Seo Young Park, A-yeon Son, Se Jin Choi, Jooae Choe, Hye Young Choi, Hye Jeon Hwang, Han Na Noh, Joon Beom Seo, Sang Min Lee, Namkug Kim

**Affiliations:** 1 Department of Medicine, University of Ulsan College of Medicine, Asan Medical Center, Seoul, Republic of Korea; 2 Department of Biomedical Engineering, Asan Medical Institute of Convergence Science and Technology, Asan Medical Center, University of Ulsan College of Medicine, Seoul, Republic of Korea; 3 Promedius Inc., Seoul, Republic of Korea; 4 Department of Statistics and Data Science, Korea National Open University, Seoul, Republic of Korea; 5 Department of Radiology and Research Institute of Radiology, University of Ulsan College of Medicine and Asan Medical Center, 88 Olympic-ro 43-gil, Songpa-gu, Seoul, 05505 Republic of Korea; 6 Department of Health Screening and Promotion Center, Asan Medical Center, Seoul, Republic of Korea; 7 Department of Convergence Medicine, University of Ulsan College of Medicine, Asan Medical Center, 88 Olympic-ro 43-gil, Songpa-gu, Seoul, 05505 Republic of Korea

**Keywords:** Diseases, Medical research, Engineering

## Abstract

The generative adversarial network (GAN) is a promising deep learning method for generating images. We evaluated the generation of highly realistic and high-resolution chest radiographs (CXRs) using progressive growing GAN (PGGAN). We trained two PGGAN models using normal and abnormal CXRs, solely relying on normal CXRs to demonstrate the quality of synthetic CXRs that were 1000 × 1000 pixels in size. Image Turing tests were evaluated by six radiologists in a binary fashion using two independent validation sets to judge the authenticity of each CXR, with a mean accuracy of 67.42% and 69.92% for the first and second trials, respectively. Inter-reader agreements were poor for the first (κ = 0.10) and second (κ = 0.14) Turing tests. Additionally, a convolutional neural network (CNN) was used to classify normal or abnormal CXR using only real images and/or synthetic images mixed datasets. The accuracy of the CNN model trained using a mixed dataset of synthetic and real data was 93.3%, compared to 91.0% for the model built using only the real data. PGGAN was able to generate CXRs that were identical to real CXRs, and this showed promise to overcome imbalances between classes in CNN training.

## Introduction

The recent rapid development of artificial intelligence in medicine can be primarily attributed to advances in algorithms, the computing power of graphics processing units (GPUs), and the generation of healthcare bigdata^1^. The number of studies^2^ applying deep learning techniques to medical imaging has increased significantly in recent years. Specifically, applications include lesion detection, image segmentation, classification, and image reconstruction^3,4^. However, there are several limitations, including a strong imbalanced dataset for specific diseases, expensive labels, and legal and ethical issues regarding patients’ privacy concerns, in implementing deep learning techniques in medical imaging^3,5^. Given the rarity of some diseases and high dependency on vast amounts of good-quality labelled data, which requires considerable time input from experts and correspondingly high expenses, many medical datasets suffer from class imbalance and insufficient labeling^6,7^. Moreover, most supervised learning models exhibit optimal performance for specific tasks in narrow clinical settings, which in turn indicates “weak” artificial intelligence. However, the technique can be ineffective with limited coverage when used in real clinical settings, such as emergency departments, because of the diversity of clinical situations and imbalanced diseases. A potential approach for overcoming these issues involves applying unsupervised learning models for many tasks, including generating synthetic dataset and, anomaly detection.

Generative adversarial networks (GANs) are effective non-supervised learning method^8,9^ that have gained popularity for their high performance in creating realistic images^10^. The generation of realistic medical images can lead to new opportunities for solving the problems involving class imbalance, data augmentation, and patients’ privacy concerns^5,8,9,11^. GANs have been utilized in various medical imaging tasks to resolve the aforementioned problems with promising results^12–18^. Additionally, in some studies, attempts have been made to boost the performance of lesion detection by subtracting the most similar and GAN-generated normal image from a pathological real image^19,20^. Despite these promising results, the generation of synthetic medical images that are realistic to the maximum extent is a prerequisite for applying GANs to actual clinical settings. However, there is a scarcity of studies in which the evaluation of the perceived “realism” of GAN-generated medical images by radiologists is assessed^21–23^. Moreover, there have been no such studies focusing on the high-resolution chest radiographs (CXRs) using GANs. Recently, a progressive growing GAN (PGGAN)^24^ was suggested in computer vision^12,21,25–29^. In some studies, a PGGAN model showed that synthetic body computed tomography images 512 × 512 pixels in size were highly realistic^21^ and the generated cephalogram X-ray images could be helpful for training convolutional neural networks (CNN) in imbalanced dataset^12^.

Therefore, we used a PGGAN to generate highly realistic CXRs and performed image Turing tests and downstream tasks for classifying normal and abnormal CXRs. The classification was used to augment the realism of the synthetic images for evaluating the model. The contributions of our study are as follows:

1. We proposed a training method for producing highly realistic and high-resolution (1000 × 1000) synthetic CXR images with GAN.
2. Six thoracic radiologists evaluated these synthetic CXR images by visual Turing test.
3. The synthetic datasets may be used to train a downstream task to classify normal or abnormal CXR images without decreasing accuracy, which in turn can be used as an augmentation technique to overcome data imbalances.

## Results

### Results of the visual Turing test

Table 1 summarizes the results of the first Turing test with ABN-PGGAN by the six readers. The mean accuracy, sensitivity, and specificity of the six readers were 67.4%, 57.3%, and 77.5%, respectively. Table 2 summarizes the results of the second Turing test with NOR-PGGAN by the six readers. The mean accuracy, sensitivity, and specificity of the six readers were 69.9%, 65.2%, and 74.7%, respectively. Inter-reader agreements of six radiologists were poor for first and second image Turing tests wherein the Kappa values (95% CI) were 0.10 (0.07–0.14) and 0.14 (0.10–0.18), respectively.

**Table 1:** Performance of the first image Turing test.

Reader	Accuracy (%, 95% CI)	Sensitivity (%, 95% CI)	Specificity (%, 95% CI)
R01	46.00 (38.95, 53.17)	59.00 (48.71, 68.74)	33.00 (23.92, 43.12)
R02	56.50 (49.33, 63.48)	76.00 (66.43, 83.98)	37.00 (27.56, 47.24)
R03	48.00 (40.90, 55.16)	59.00 (48.71, 68.74)	37.00 (27.56, 47.24)
R04	89.50 (84.40, 93.38)	90.00 (82.38, 95.10)	89.00 (81.17, 94.38)
R05	73.50 (66.81, 79.48)	86.00 (77.63, 92.13)	61.00 (50.73, 70.60)
R06	91.00 (86.15, 94.58)	95.00 (88.72, 98.36)	87.00 (78.80, 92.89)

**Table 2:** Performance of the second image Turing test.

Reader	Accuracy (%, 95% CI)	Sensitivity (%, 95% CI)	Specificity (%, 95% CI)
R01	52.00 (44.84, 59.10)	63.00 (52.76, 72.44)	41.00 (31.26, 51.29)
R02	52.50 (45.34, 59.59)	59.00 (48.71, 68.74)	46.00 (35.98, 56.26)
R03	90.50 (85.56, 94.18)	96.00 (90.07, 98.90)	85.00 (76.47, 91.35)
R04	83.00 (77.06, 87.93)	67.00 (56.88, 76.08)	99.00 (94.55, 99.97)
R05	50.50 (43.36, 57.63)	67.00 (56.88, 76.08)	34.00 (24.82, 44.15)
R06	91.00 (86.15, 94.58)	96.00 (90.07, 98.90)	86.00 (77.63, 92.13)

As shown in Table 3, more experienced readers exhibited a higher probability of guessing the correct answer in the first Turing test dataset. However, there was no statistical difference between two groups in the second test dataset. As shown in Table 4, the reading time was higher when readers answered correctly in only synthetic images irrespective of whether the correction for the reader effect was considered. However, it was observed that the reading time was shorter when the reader effect was corrected in only real images.

**Table 3:** Mixed effect logistic regression model for correcting answers in the image Turing test.

Datasets	Applied data	Odds ratio of correct answer (95% CI)	p-value
First Turing test dataset	All	6.65 (3.29, 13.47)	< 0.001
	Only real images	5.30 (2.75, 10.22)	< 0.001
	Only synthetic images	7.69 (3.29, 17.98)	< 0.001
Second Turing test dataset	All	1.67 (0.33, 8.42)	0.54
	Only real images	1.22 (0.17, 8.90)	0.85
	Only synthetic images	3.95 (0.31, 51.34)	0.29

Regression model: logit of correct answer ~ expert or novice + image random effect + reader random effect.

**Table 4:** Mixed effect logistic regression model for reading time in the image Turing test.

Datasets	Applied data	Regression model 1		Regression model 2	
		Beta coefficient of reading time (95% CI)	p-value	Beta coefficient of reading time (95% CI)	p-value
First Turing test dataset	All	− 0.027 (− 0.088, 0.033)	0.376	0.202 (0.125, 0.278)	< 0.001
	Only real images	− 0.196 (− 0.283, − 0.109)	< 0.001	0.105 (− 0.017, 0.227)	0.091
	Only fake images	0.090 (0.003, 0.177)	0.044	0.277 (0.175, 0.379)	< 0.001
Second Turing test dataset	All	− 0.052 (− 0.131, 0.027)	0.195	0.074 (− 0.002, 0.150)	0.058
	Only real images	− 0.003 (− 0.116, 0.111)	0.962	0.127 (0.015, 0.238)	0.027
	Only fake images	− 0.024 (− 0.137, 0.089)	0.674	0.020 (− 0.084, 0.124)	0.706

Regression model 1: log(reading time + 1) ~ correct (y/n) + image random effect + reader random effect.

Regression model 2: log(reading time + 1) ~ correct (y/n) + image random effect.

**Figure 1:**
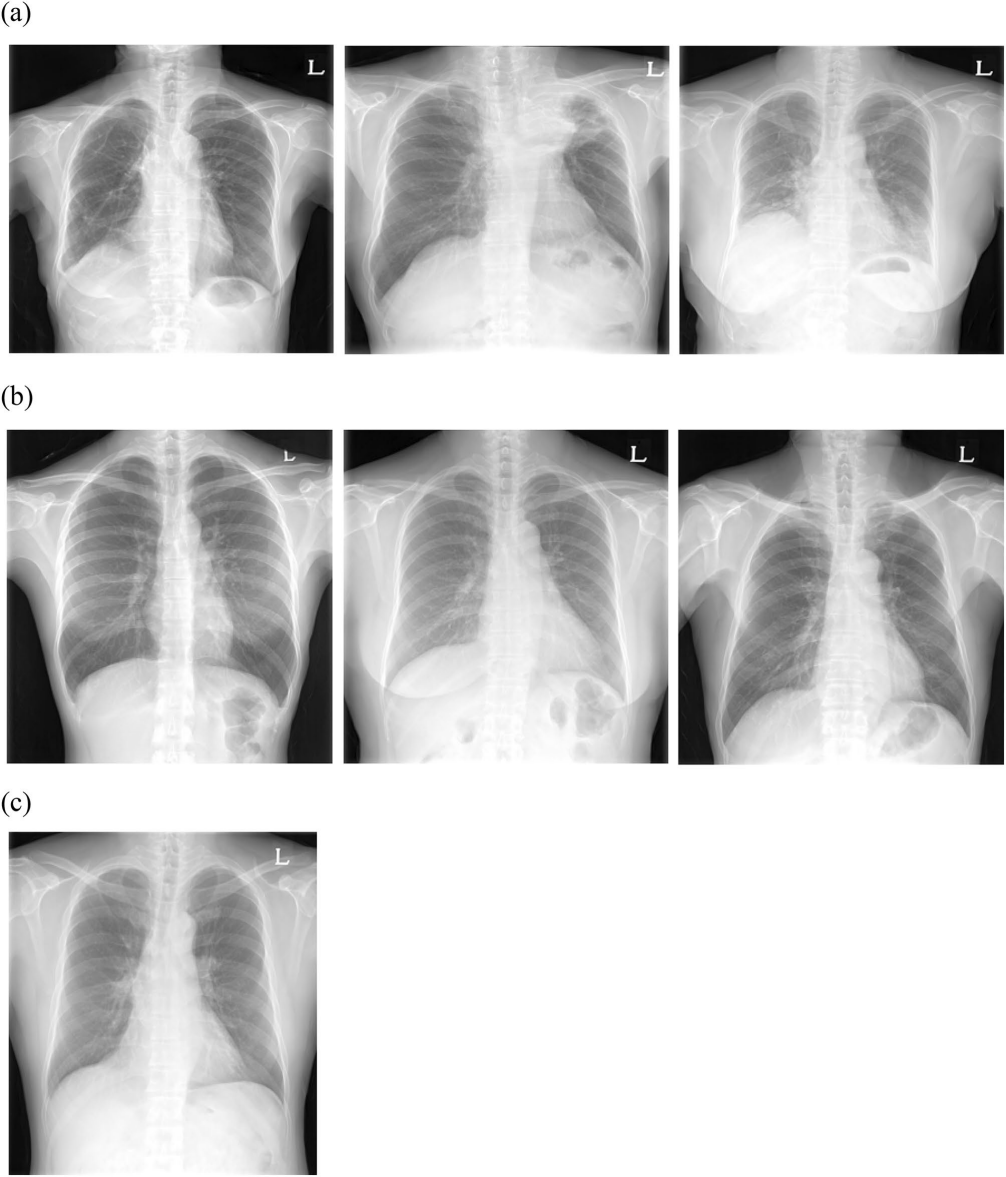
Several cases of the Turing test. (**a**) Synthetic images wherein the decisions of all the readers are synthetic in the first Turing test. (**b**) Synthetic images wherein the decisions of all the readers are synthetic in the second Turing test. (**c**) Synthetic images wherein the decisions of all the readers are real in the first Turing test.

As shown in Fig. 1, six synthetic images were judged as synthetic by all the readers and one synthetic image was judged as real by all the readers.

### Normal probability scores in real and synthetic images and Grad-CAM

**Figure 2:**
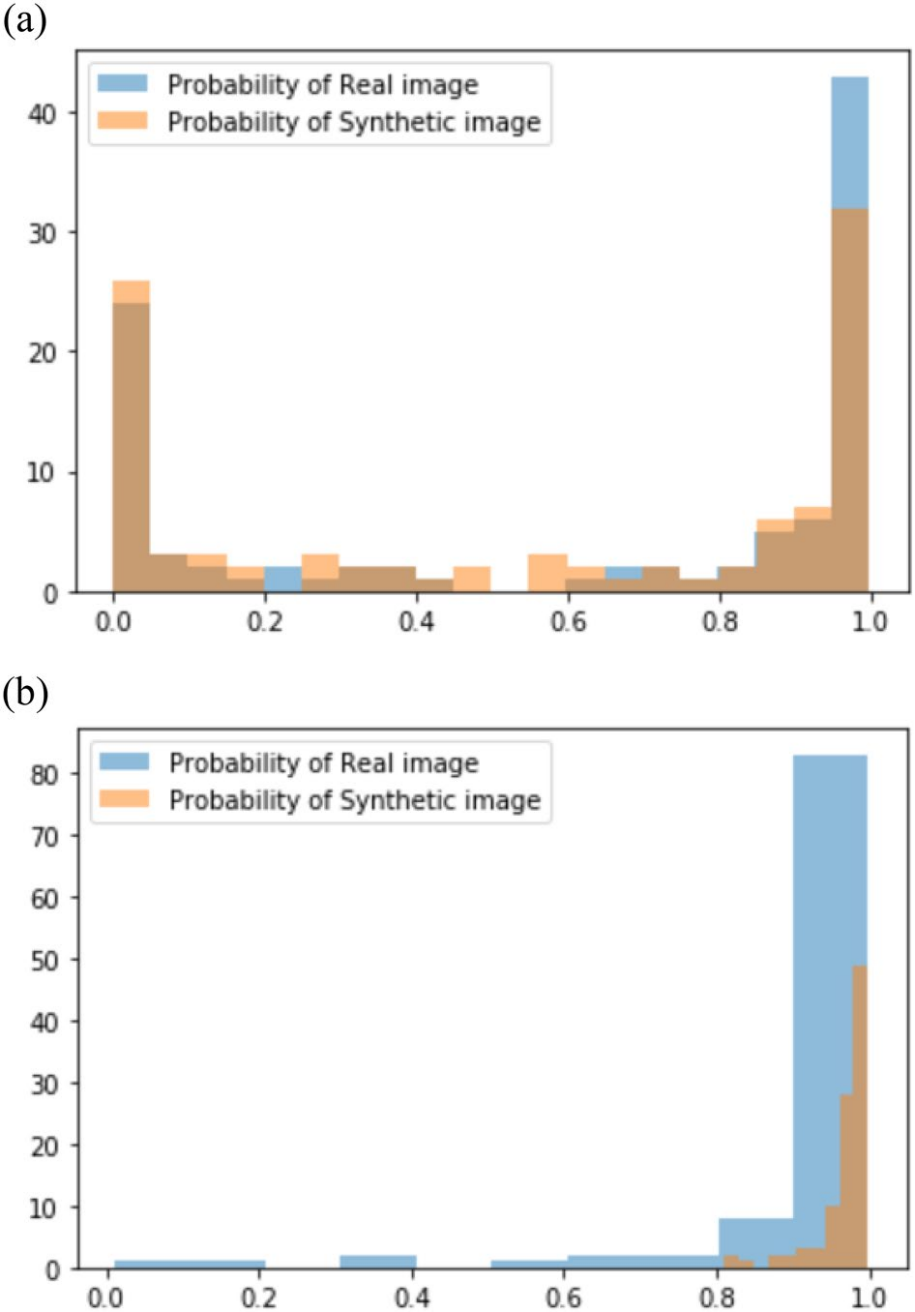
Probability scores of the CNN classifier in Turing test datasets. (**a**) First image Turing test dataset. (**b**) Second image Turing test dataset.

**Figure 3:**
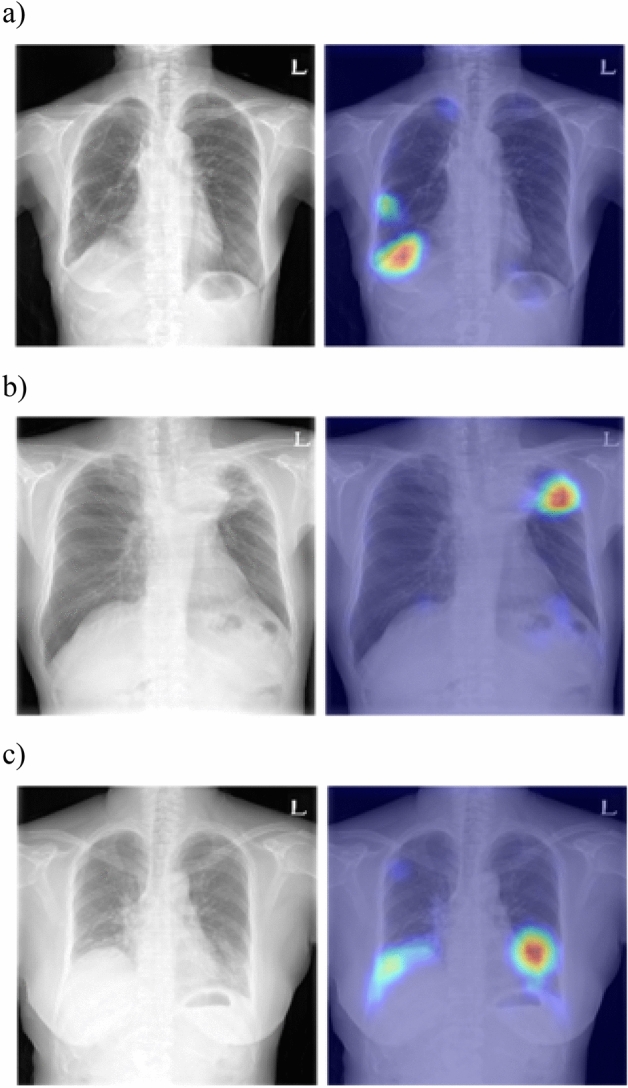
Grad-CAMs of synthetic images in the first Turing test dataset wherein the decisions of all readers are synthetic.

Figure 2 shows the normal probability scores of the known CNN classifier with real and synthetic images in the abnormal dataset (a) and normal dataset (b). Additionally, the Grad-CAMs of synthetic images that decisions of all readers were “synthesized” are shown in the Fig. 3. The Grad-CAMs highlighted abnormal lung lesions in three synthetic images, which were not considered real by all the readers.

### Performances of models using real and synthetic mixed datasets.

**Figure 4:**
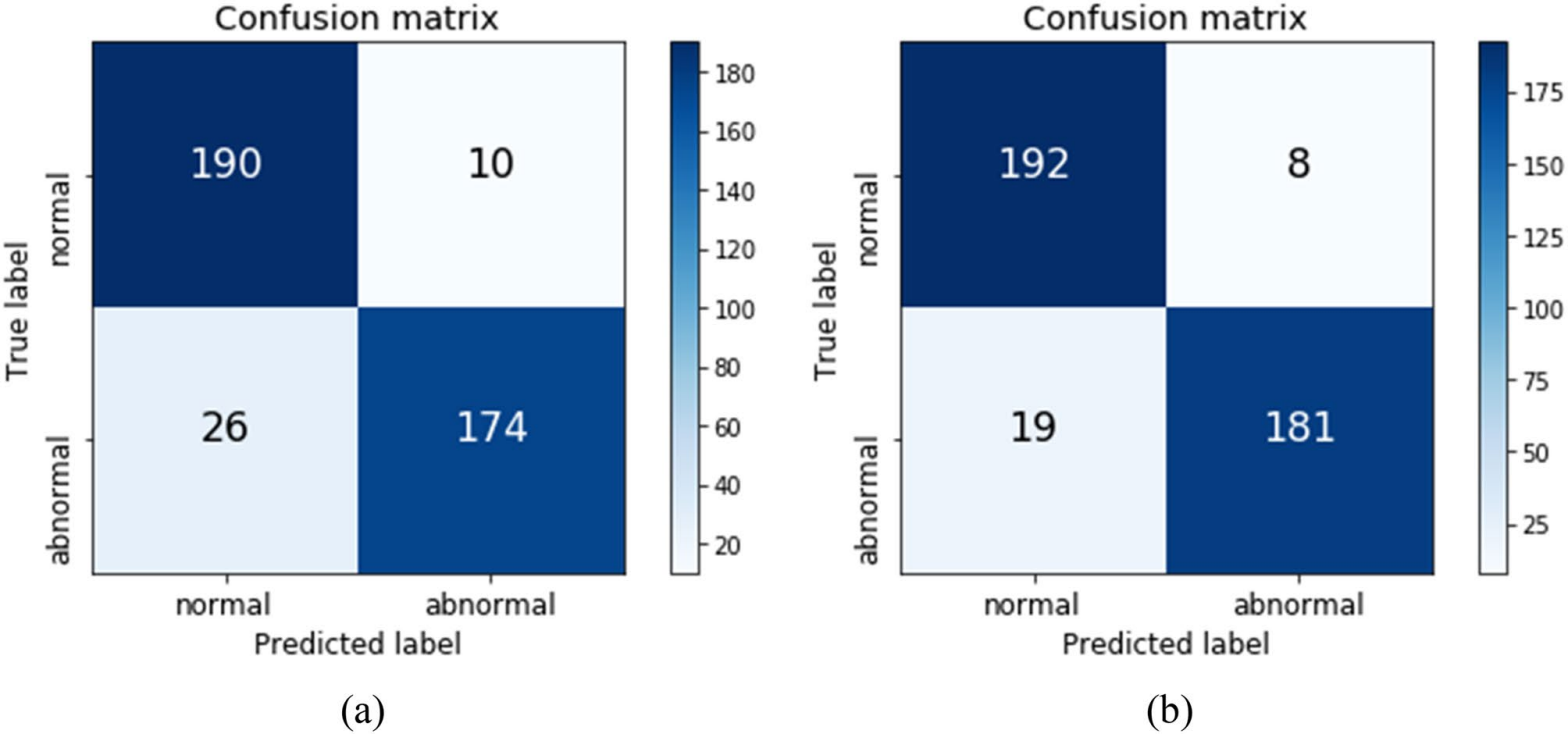
Confusion matrices of the two models. (**a**) Performance of the trained model using only the real dataset. (**b**) Performance of the trained model using the synthetic mixed dataset.

Figure 4 shows the confusion matrices of the two models. The AUROCs of the same real test set were 0.96 and 0.98 for the models trained using the real and synthetic mixed datasets, respectively. For the model trained using the real dataset, the overall accuracy was 91.0%, sensitivity was 87.0%, and specificity was 95.0%, while for the model trained using the synthetic mixed dataset, the overall accuracy was 93.3%, sensitivity was 90.5%, and specificity was 95.6%. The results of supplemental experiments, which were performed with more than 6000 images of training data, were similar. The AUROCs of the same real test set were 0.98 and 0.98 for the models trained using the real and synthetic mixed datasets, respectively. The confusion matrices are presented in supplement Fig. S1.

## Discussion

In this study, we demonstrated the generation of high-resolution CXRs using PGGAN and two visual Turing tests of synthetic and real CXRs of abnormal and normal patients performed by six readers with different experience levels. The test results indicate that readers, excluding most experienced radiologists exhibited different performances in terms of sensitivity and specificity, with extremely low Kappa values in the two Turing tests^30^. Therefore, the radiologists were not able to distinguish synthetic CXRs from real CXRs. The first step in applying GAN to the development of models applicable to medical fields depends on the generation of highly realistic and high-resolution images. At the beginning of the study, we questioned whether realistic images 1024 × 1024 pixels in size can be generated by ABN-PGGAN and NOR-PGGAN. Although several reports have investigated the benefits of GANs in various medical imaging fields^7,9,31,32^, evaluation of the realistic nature of GAN-generated synthetic images via a comparison of normal and abnormal CXRs has not been reported to the best of the author’s knowledge. Therefore, the realistic nature of synthetic images has not been well validated.

There were different points between the two Turing tests. Odds ratios in the first Turing test statistically exceeded those in the second Turing test in correcting answers, specifically when only synthetic images were used, thereby indicating that synthetic images generated from ABN-PGGAN were less real. Nevertheless, the results of beta coefficient on reading time of abnormal images in the first Turing test suggested it was difficult to judge synthetic images as synthetic. Furthermore, more time was required to obtain the correct answer in the first Turing test when compared to that in the second Turing test.

In this study, radiologists were not able to distinguish normal synthetic CXRs from real CXRs, irrespective of their expertise; however, more experienced radiologists were able to differentiate abnormal synthetic chest CXRs from abnormal synthetic CXRs, whilst ABN-PGGAN trained more CXRs than NOR-PGGAN. However, the range of abnormal CXRs is suspected to vary significantly when trained by GAN. Therefore, more experienced readers can detect artificial findings with abnormal lesions in synthetic images. In the analysis of reading time, when considering reader effect, reading time was short when readers determined real images as real in the first Turing test dataset. Conversely, NOR-PGGAN trained normal CXRs features relatively well. In the present study, inter-reader agreement was poor for the entire image set consisting of synthetic and real images, which indicates that it is difficult to distinguish between synthetic and real images (i.e., identifying realistic synthetic images).

In CXRs, several lines and stripes are observed from the anatomic structures in the chest. Radiologists are trained to recognize their normal and abnormal appearances of lines and stripes on CXRs. In certain synthetic images, some discontinuities are present in the lines and stripes including interface of lung, vessels, and ribs, this tends to be more pronounced in abnormal synthetic images. By competing for the generator and discriminator, the GAN converges to an appropriate local minimum, and the generator produces a realistic image with a given latent vector^33^. The latent space of GANs is the result of learning the mapping from a latent distribution to the real images via adversarial training^34^. The latent spaces of ABN-PGGAN included pathologic features and less normal features when compared with those of NOR-PGGAN.

From the CNN viewpoint, the CNN was unable to distinguish between synthetic and real images, with the synthetic images considered more appropriate for training based on the purpose. All normal synthetic images generally exhibited normal probability scores of close to 1. The borderline of normal and abnormal CXRs was not evident, and thus the ABN-PGGAN trained dataset included normal CXRs. Furthermore, the visual Turing test of the abnormal dataset included normal real CXRs. The probability scores in the visual Turing test of abnormal dataset varied. However, the Grad-CAMs of abnormal synthetic images, which all readers determined as fake, highlighted pathologic lesions as opposed to artificial regions.

Furthermore, synthetic images were used to develop the classification task. The effects of class imbalance on the performance of CNNs were examined, where it was determined to decrease the performance of CNN^35,36^. The CNN model was trained on two datasets, namely only real and mixed synthetic datasets. There is always a shortage of disease data in clinical situation, meaning that the mixed synthetic dataset included abnormal synthetic CXRs. The classification performance of two models were comparable and synthetic images improved the performance of CNN classifiers. In general, 1000 images were used as a rough criterion for training a model per class. In a recent study, approximately 50,000 images per class was deemed necessary for acceptable performance^37^. In our current study, enough images were generated for further studies. Additionally, disentangling the latent space of GAN leads to controllable abnormal CXR features^38^, indicating that synthetic images generated by GAN can lead to a solution for training CNN in rare diseases. Since a method for evaluating the quality of the generated data has not yet been established, when synthetic data alone are used, performance may be lower than when using real data.

The potential clinical applicability of useful GANs can be image reconstruction and denoising^16,39^, translation between different radiologic modalities^14,40,41^, and anomaly detection^19,29^. Detecting abnormalities is predicated on learning the probability distribution of normal training data, unlike other GAN-based tasks. Any image data that deviate from this distribution are regarded as abnormal. In daily clinical situations, diagnostic images are clinically acquired for patients with a variety of diseases. If GAN models can filter out normal chest x-rays well, doctors can focus more on chest images with abnormal findings. However, since the normal range may vary depending on the clinical situation and the age of the patient, many additional studies are needed to actually utilize it.

Although several evaluation metrics were used to measure the quality of generated images using GAN models, such as Inception Score (IS)^42^ or Fréchet Inception Distance (FID)^43^, the metrics did not fully explain the extent of failure or success of the generated synthetic images in medical images. This is because the IS and FID metrics solely focus on the distributions of synthetic images using a CNN network (Inception V3), as well as ignore the semantics of the images. Therefore, it was concluded that, to date, a visual Turing test by human experts is the only viable solution to fairly evaluate the quality of generated synthetic medical images.

This study included several limitations. First, for ABN-GAN training, the ratio of abnormal and normal CXRs was approximately 8:2, which was emphatically determined. However, it is necessary to further train the model to generate more realistic abnormal images. Second, there are various types of GAN architectures including StyleGAN2^44^, StyleGAN2-Adaptive Discriminator Augmentation (ADA)^45^, and score-based generative model^46^, which can be evaluated in this manner. We have used StyleGAN2-ADA model^45^ for training with the learning rate of 0.002 and r1_gamma of 26.2144 without mirror augmentation. The details of generated CXRs seem to fall off in minute parts such as ribs and pulmonary blood vessels until now. Further experiments and research are currently ongoing. Third, given the need to further examine the latent space of GAN proceeds, additional research must focus on determining the amount of and range of training data. Finally, the number of readers of the visual Turing test was relatively low, meaning that it is not possible to generalize factors related to the experience of readers. Hence, this should be examined further.

In conclusion, ABN- and NOR-PGGAN models were able to generate highly realistic and high-resolution CXRs that were validated by radiologists with different levels of expertise and a previously trained CNN classifier. The generation power of NOR-PGGAN was considered to exceed that of ABN-PGGAN, indicating that GAN generated abnormal images require more data. Nevertheless, the proposed models imply significant value for the development of CNN models using GAN-based data augmentation. Further research will also be able to show the utilization of GAN-generated data for developing anomaly detection for avoiding expensive labels, overcoming strong imbalanced datasets for rare diseases, and avoiding legal and ethical issues related to privacy concerns.

## Materials and methods

### Ethical approval

This retrospective study was conducted according to the principles of the Declaration of Helsinki and in accordance with current scientific guidelines. The study protocol was approved by the Institutional Review Board Committee of Asan Medical Center (AMC), Seoul, Korea (No. 2019-0321). The requirement for informed patient consent was waived by the Institutional Review Board Committee of Asan Medical Center because of the retrospective nature of this study.

### Data collection

**Figure 5:**
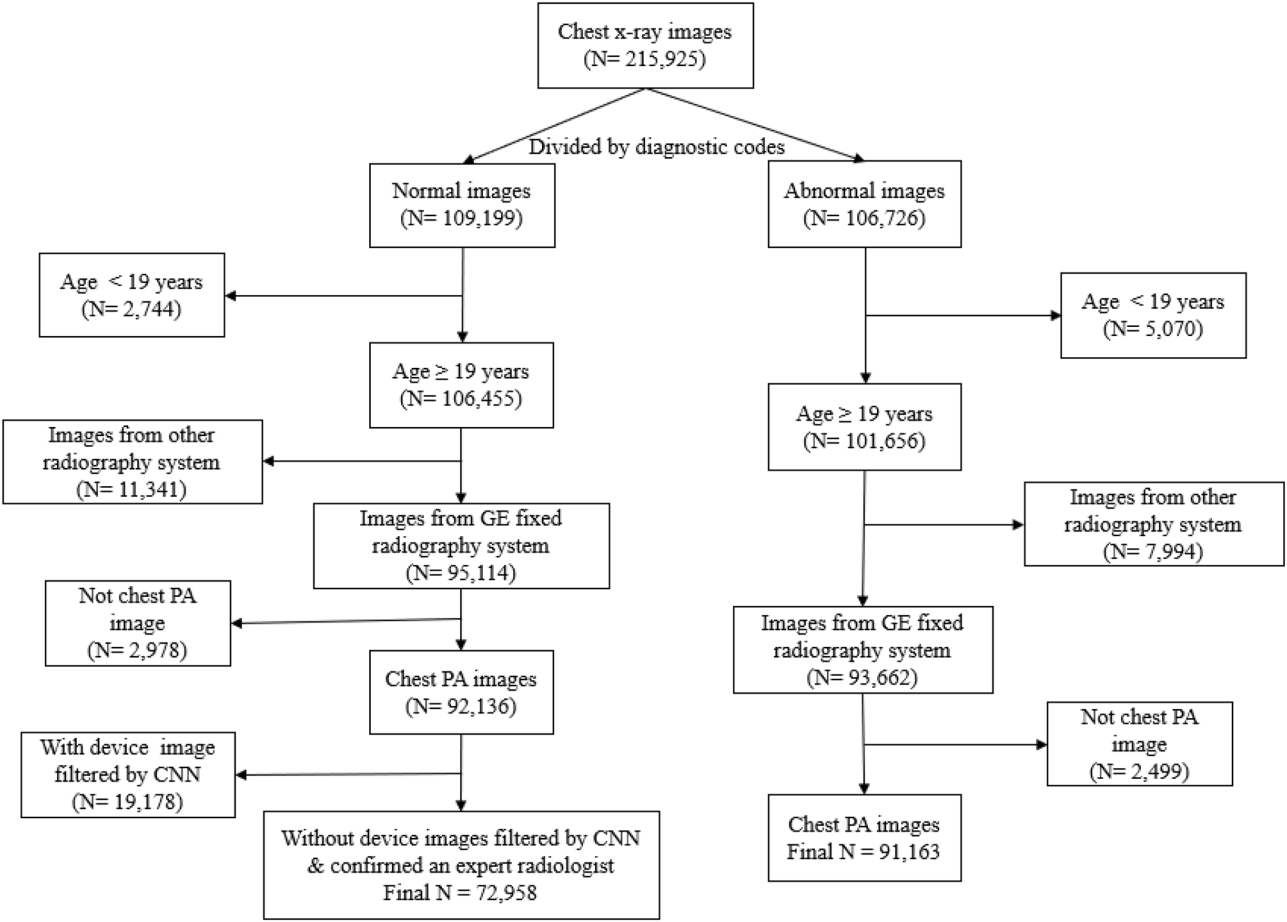
Overview of collection of normal and abnormal datasets collection.

A large number of chest X-ray images were collected in the department of radiology of AMC between January 2011 and December 2018. The original dataset was cleaned as illustrated in Fig. 5. Normal and abnormal chest x-ray images were classified using diagnostic codes. This study was conducted on chest x-rays of adults aged 19 years and older, and the images were included solely from fixed radiography systems of GE Healthcare. The age range generally agreed upon as an adult was selected because body parts included in chest X-ray images may vary due to differences in body size according to age, and differences in prevalent diseases also exist in children and adolescents. This ensured the control of domain shift due to various types of x-ray equipment. Furthermore, CXR posteroanterior (PA) images were acquired by removing other chest images because the original dataset contained various types of chest images, such as chest lateral images and chest decubitus images, which can be only differentiated by using DICOM fields. For selecting a normal CXR PA image, classified normal CXRs with many devices or wires, such as central venous catheters and ECG lines, were excluded from the normal group via the simple convolutional neural network (CNN) classifier. This was further confirmed by an expert radiologist. Finally, the number of normal group chest images was 72,958 and abnormal group chest images was 91,163. The DICOM files of CXRs were converted into 1024 × 1024 pixel-sized 8-bit PNG format with normal or abnormal labels.

### Training PGGAN models

PGGAN is a GAN variant and consists of two networks including a generator and discriminator. The PGGAN model was selected to generate high resolution CXRs because this model exhibits better performance in reconstructing a global structure and fine details with high resolution when compared with other GAN variant models^47–49^. Furthermore, PGGAN learns to generate images starting from a low resolution of 4 × 4 pixels to a high resolution of 1024 × 1024 pixels, by progressively growing generator and discriminator networks^24^. The general characteristics of the training images are trained through progressive learning and detail-oriented characteristics are trained in addition to layer growth. The output of the lower resolution layer has an impact on the high-resolution output due to the fade-in type used while raising resolution. PGGAN gradually creates a high-resolution image from a large-scale low-resolution image while considering the learning outcome of a previous layer. A publicly available official website of PGGAN was implemented using TensorFlow in Python (Tensorflow-gpu 1.6.0, Python 3.4.0). GAN training is defined by a game theory in which two players compete against each other. The generator network learns to map a noise to the input space, and the discriminator network learns to distinguish between the generated and true samples. Formally, the loss function is defined by minimax objective:

$$\underset{\mathrm{G}}{\mathrm{min}}\underset{\mathrm{D}}{\mathrm{max}} \underset{\mathrm{x}\sim {\mathbb{P}}_{r}}{\mathbb{E}}[\mathrm{log}\,\,D(x)] + \underset{\widetilde{\mathrm{x}}\sim {\mathbb{P}}_{g}}{\mathbb{E}}[\mathrm{log}(1 -D(\widetilde{x}))]$$

where ${\mathbb{P}}_{r}$ is the data distribution of images (real), ${\mathbb{P}}_{g}$ is the model distribution implicitly defined by $\widetilde{x} =\mathrm{ G}(\mathrm{z})$, $z\sim p\left(z\right)$ ($P(z)$ is Gaussian distribution), and ${\mathbb{P}}_{\widehat{x}}$ is defined by uniformly sampled along straight lines between point pairs sampled from ${\mathbb{P}}_{r}$ and ${\mathbb{P}}_{g}.$

PGGAN use the improved Wasserstein GAN loss^50^ (WGAN-GP loss), which perform better than Wasserstein GAN (WGAN)^51^ by virtue of gradient penalty.

In this study, we selected PGGAN with the improved Wasserstein GAN (WGAN-GP) loss, as it stabilizes the training sufficiently to synthesize high-resolution images. The equation of WGAN-GP loss is defined as:

$$\mathit{L }= \underset{\widetilde{\mathrm{x}}\sim {\mathbb{P}}_{g}}{\mathbb{E}}\left[D\left(\widetilde{x}\right)\right]-\underset{x\sim {\mathbb{P}}_{r}}{\mathbb{E}}\left[D\left(x\right)\right] + \lambda \underset{\widehat{x}\sim {\mathrm{\rm P}}_{\widehat{x}}}{\mathbb{E}}\left[{\left({\Vert {\nabla }_{\widehat{\mathrm{x}}}D\left(\widehat{\mathrm{x}}\right)\Vert }_{2} - 1\right)}^{2}\right]$$

A value of $\lambda = 10.0$ was used in the experiments.

In this study, the PGGAN model was trained with 91,163 abnormal CXRs and randomly sampled 20,000 normal CXRs as abnormal-PGGAN (ABN-PGGAN) for training spectrum from normal to abnormal CXRs. Because there is a region in which the boundary between normal and abnormal cannot be clearly divided. Furthermore, the PGGAN model was trained solely with 72,958 normal CXRs as normal-PGGAN (NOR-PGGAN) as a control study for generating normal CXRs. The variations in normal CXRs were regarded as smaller than those of abnormal CXRs. ABN-PGGAN was trained for 130 epochs and required approximately 12.2 days with two Nvidia Titan RTX GPUs, and NOR-PGGAN was trained for 160 epochs, which required approximately 12.5 days with two Nvidia P40 GPUs. Additionally, two distinct sets of 50,000 synthetic CXR images were generated by using the trained generators of ABN-PGGAN and NOR-PGGAN.

### Image Turing test

The image Turing test was conducted twice to assess the realistic nature of synthetic CXRs, specifically, 400 CXRs were selected for the test. Fifty percent of these CXRs were randomly selected from the two sets of 50,000 synthesized images while the other fifty percent of the images were real images that were randomly selected from the training set. The first set of Turing test images consisted of 100 real normal and abnormal chest images, randomly sampled from ABN-PAGGAN trained set, and 100 synthetic images randomly sampled from 50,000 images generated by ABN-PGGAN. The second set of Turing test images consisted of 100 real normal chest images, randomly sampled from NOR-PAGGAN trained set, and 100 synthetic images randomly sampled from 50,000 images generated by NOR-PGGAN.

To avoid selection bias, not all the synthetic images were individually selected by the researchers. The image Turing test was conducted with six readers (radiology residents and four thoracic radiologists) by displaying images one-by-one via a web-based interface (supplement Fig. S5). The readers comprised of one-year and three-year radiology residents and one-year, three-year, ten-year, and twenty-year radiology specialists. To reduce environmental variability during the Turing test, the images were displayed in the same order, and any previous answers could not to be modified. All readers successfully performed the test and decided whether each image was real or synthetic without any time limit and no prior information on the number of real or synthetic images. Additionally, sensitivity, specificity, accuracy and reading time were derived after the image Turing tests were completed.

### Comparison of the CNN classifier on real and synthetic images

We measured the normal probability score of the 200 synthetic and 200 real images by using our previously trained classifier with an accuracy of 94.7 to differentiate between normal and abnormal CXRs^52^. As the probability score tends to 1, the probability of a normal CXR increases. To determine the decision-making process of the model and identify the most important regions of the model for classifying abnormal CXRs in the abnormal dataset, the gradient-weighted class activation mapping technique (Grad-CAM)^53^ was used by overlaying the most significant regions of abnormal lesions in the images with red color.

### Efficacy of synthetic images by comparing the performance of models trained solely on real and/or synthetic mixed datasets

To verify the utility of the synthetic images, a CNN-based classification was performed as a downstream task. The task involved classifying normal or abnormal images in only the real chest radiographs dataset and adding synthetic images generated by ABN–PGGAN. The synthetic images were added, because normal images significantly exceeded abnormal chest radiographs in the real world.

Therefore, the real dataset consisted of 1000 normal and 1000 abnormal chest radiographs, wherein the latter included 200, 200, 200, 200, and 200 images with nodules, consolidation interstitial opacity, pleural effusion, and pneumothorax, respectively^52^. Normal and abnormal datasets with nodule[s], including mass/consolidation or interstitial opacities, were confirmed via chest CT and pleural effusion. Furthermore, pneumothorax on CXRs were determined via consensus of two thoracic radiologists with corresponding chest CT images. The real dataset was randomly split into 80% for training and 20% for testing. The test set was fixed, and half of the abnormal chest radiographs from the training dataset were randomly sampled for use in the synthetic mixed dataset.

The synthetic mixed dataset was composed of 800 normal and 800 abnormal chest radiographs. Specifically, 800 normal chest radiographs were from the training dataset of the real dataset, 400 abnormal chest radiographs were from the training dataset of the real dataset, and 400 abnormal chest images were ABN-PGGAN-generated images with high abnormal probability score according to the known CNN classifier^52^.

In addition, supplemental experiments were conducted with the more training dataset than the previous training dataset using 3269 normal and 3269 patients with including 904, 510, 240, 1324, and 291 CXRs with nodules, consolidation, interstitial opacity, pleural effusion, and pneumothorax, respectively^44^. The synthetic mixed dataset was constructed by including only 1635 synthetic abnormal images from the abnormal data and subtracting the same number of real data. The 1635 synthetic images were randomly extracted from 50,000 images generated by ABN-PGGAN.

The training using real and synthetic mixed datasets was performed via vanilla ResNet-50^54^, which was chosen as a baseline for training classification models and its performance was excellent. To compare performance, two models were tested on the fixed real test set, and an area under the receiver operating characteristic curve (AUROC) was drawn.

### Statistical analysis

The sensitivity, specificity, accuracy, and reading time of the six readers were calculated for the image Turing test. Inter-reader agreement was evaluated using Fleiss Kappa. The 95% confidence intervals (CI) of accuracy, sensitivity, and specificity were computed using binomial distribution^55^. To investigate how experience influences the probability of the correct answer, readers were classified into two groups based on their work experience. Specifically, R04, R05, and R06 were part of a more-experienced group and R01, R02, and R03 were part of a less-experienced group. Given that the results of the image Turing test were in a binary format for each image and the data were correlated with each individual and each image, mixed effect logistic regression, which models each reader and each image as random effects, was used to test whether the more-experienced group exhibited higher probability for correct answers than the less-experienced group. To evaluate the effect of experience on reading time, linear mixed models were used with each image and with/without each reader as random effects^56^.

## Supplementary Information

Supplementary Information.
