# Supplementary material for: Image Turing test and its applications on synthetic chest radiographs by using the progressive growing generative adversarial network
Source: Sci Rep. 2023 Feb 9;13:2356. doi: 10.1038/s41598-023-28175-1 (PMC9911730; doi:10.1038/s41598-023-28175-1)
Supplement: Supplementary file 1 — Supplementary Information. [file 41598_2023_28175_MOESM1_ESM.docx]

**Title:** Image Turing Test and Its Applications on Synthetic Chest Radiographs by using the Progressive Growing Generative Adversarial Network

**Miso Jang**^1,2^**, MD, PhD; Hyun-jin Bae**^3^**, PhD; Minjee Kim**^3^**, MS; Seo Young Park**^4^**, PhD; A-yeon Son**^5^**, MD; Se Jin Choi**^5^**, MD; Jooae Choe**^5^**, MD, PhD; Hye Young Choi** ^5^**, MD; Hye Jeon Hwang** ^5^**, MD, PhD; Han Na Noh**^6^**, MD; Joon Beom Seo**^5^**, MD, PhD; Sang Min Lee**^5^***, MD; Namkug Kim**^7,8^***, PhD.**

**AFFILIATIONS:**

*^1^Department of Medicine, University of Ulsan College of Medicine, Asan Medical Center, Seoul, Republic of Korea*

*^2^Department of Biomedical Engineering, Asan Medical Institute of Convergence Science and Technology, Asan Medical Center, University of Ulsan College of Medicine, Seoul, Republic of Korea*

*^3^Promedius Inc., Seoul, Republic of Korea*

*^4^Department of Statistics and Data Science, Korea National Open University, Seoul, Korea.*

*^5^Department of Radiology and Research Institute of Radiology, University of Ulsan College of Medicine & Asan Medical Center, Seoul, Republic of Korea*

*^6^Department of Health Screening and Promotion Center, department of diagnostic radiology, Asan Medical Center, Seoul, Republic of Korea*

*^7^Department of Radiology, University of Ulsan College of Medicine, Asan Medical Center, Seoul, Republic of Korea*

*^8^Department of Convergence Medicine, University of Ulsan College of Medicine, Asan Medical Center, Seoul, Republic of Korea*

***Co-corresponding authors:**

**Namkug Kim, PhD**

Associate Professor, Department of Convergence Medicine

University of Ulsan, College of Medicine, Asan Medical Center

88 Olympic-ro 43-gil, Songpa-gu, Seoul 05505, Korea

Tel: +82-2-3010-6573; Fax: +82-2-3010-6196; Email: namkugkim@gmail.com

**Sang Min Lee, MD, PhD**

Department of Radiology and Research Institute of Radiology,

University of Ulsan College of Medicine, Asan Medical Center,

88 Olympic-ro 43-gil, Songpa-gu, Seoul 05505, Republic of Korea

Tel: +82-2-3010-5766 ; Fax: +82-2-3010-6196 ; Email: asellion@hanmail.net

Supplement figure 1. The screen shot of the first webpage of image Turing test.


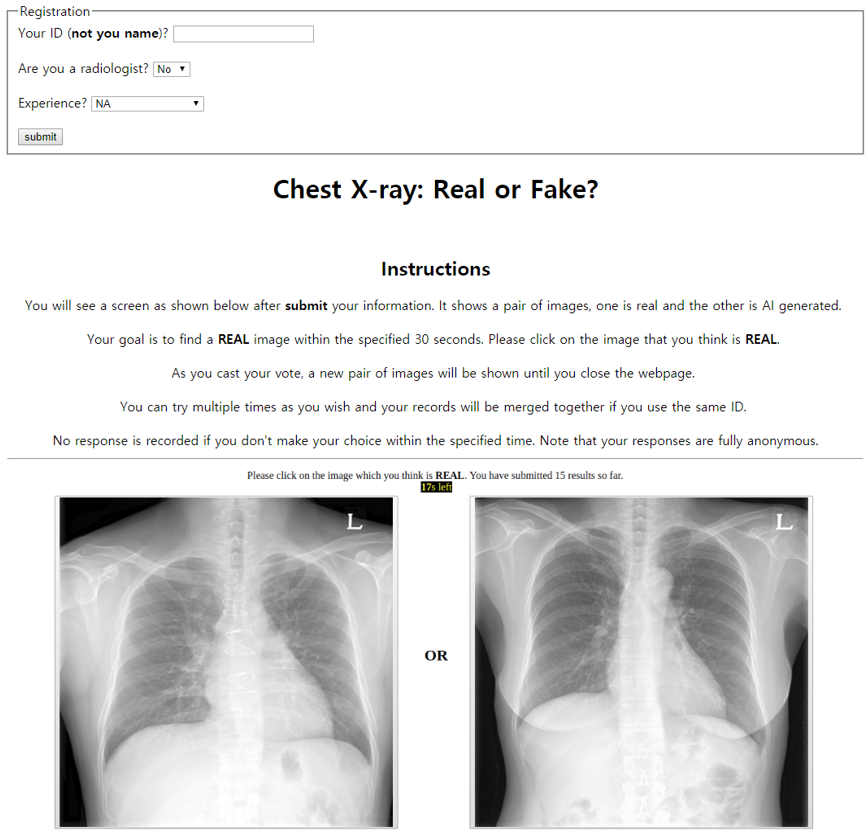


Supplement figure 2. Confusion matrices of the two models trained with more dataset.


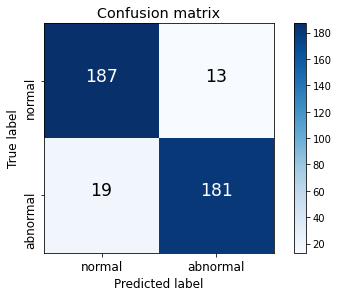

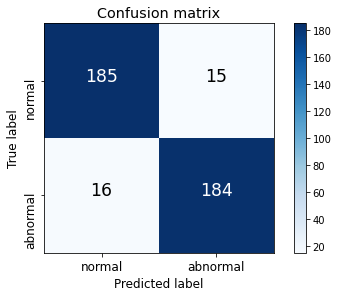


(a) (b)

(a) Performance of the trained model using only the real dataset. (b) Performance of the trained model using the synthetic mixed dataset.
